# ADAPT‐marketplace – A web‐application for XML‐based template sharing in radiation oncology

**DOI:** 10.1002/acm2.70148

**Published:** 2025-07-13

**Authors:** Dennis N. Stanley, Joseph Harms, Gaurav N. Rathi, Joel A. Pogue, Ritish Nedunoori, John B. Fiveash, Richard A Popple, Carlos E. Cardenas

**Affiliations:** ^1^ Department of Radiation Oncology University of Alabama at Birmingham Birmingham Alabama USA; ^2^ Department of Radiation Oncology Washington University School of Medicine St. Louis Missouri USA

**Keywords:** ethos, online adaptive radiotherapy, planning templates, radiation oncology, template repository, treatment planning, XML

## Abstract

**Purpose:**

The adaption of radiotherapy (RT) plans in response to anatomical and physiological changes during treatment marks a significant shift toward personalized cancer care. However, the complexity of Online Adaptive Radiotherapy (OART) procedures often leads to variability in treatment quality across institutions. The development of planning templates, particularly through the Ethos treatment planning system (TPS) and Intelligent Optimization Engine (IOE) (Varian Medical Systems, Palo Alto, CA), plays a crucial role in standardizing and streamlining OART. To address the challenges of sharing and optimizing treatment templates across diverse clinical environments, we developed the ADaptive rAdiotherapy Planning Template (ADAPT‐) Marketplace to facilitate the exchange of XML‐based templates and promote collaborative innovation in the OART community.

**Methods:**

ADAPT‐Marketplace was developed using the Django web framework, chosen for its security, scalability, and versatility. The platform supports the sharing of XML‐based treatment planning templates, compatible with Ethos TPS, and includes features for uploading, downloading, comparing, and editing templates. The development followed a structured process, involving collaborator consultations, prototyping, and both alpha and beta testing. Testing phases included predefined tasks and unscripted evaluations, with feedback collected to refine the platform prior to its official launch.

**Results:**

Since its launch in July 2023, ADAPT‐Marketplace has registered 65 users from 16 countries and 38 institutions, including academic, non‐academic and industrial sectors. Over 50 templates have been uploaded, covering treatment sites such as the pelvis, thorax, head‐and‐neck, and abdomen. Feedback from alpha and beta testing resulted in key improvements, including enhanced navigation, template validation, and user experience.

**Conclusion:**

ADAPT‐marketplace provides a centralized platform for sharing and collaborating on treatment planning templates, offering the potential to improve research productivity, facilitate knowledge exchange, and standardize OART practices across institutions.

## 1 INTRODUCTION

The adaptation of radiotherapy (RT) plans in response to anatomical and physiological changes marks a shift toward personalized care. However, the complexity of Online Adaptive Radiotherapy (OART) requires high expertise and resources, often causing variability in treatment quality across institutions. As OART adoption grows, planning templates are critical for streamlining treatment in diverse clinical settings. The Ethos treatment planning system (TPS) and Intelligent Optimization Engine (IOE) (Varian Medical Systems, Palo Alto, CA) have been instrumental in providing customizable templates. Ethos planning templates are structured XML files that encode treatment planning details such as target volumes, normal tissues, derivations, dose objectives, and optimization priorities. These templates define the parameters used by the Intelligent Optimization Engine (IOE) to generate treatment plans, ensuring consistency and reproducibility in plan quality. Importantly, Ethos templates are transferable between different Ethos systems, allowing clinics to share and implement successful treatment strategies across institutions, which promotes standardization and improved plan quality. During online adaptation with the Ethos platform, the plan optimization process follows the same template‐defined optimization scheme for each fraction. Because real‐time adjustments to the optimization are not possible during treatment appointment, the robustness of the template is critical. Strategies for defining optimization structures and derivations within the template can reduce the need for manual contour adjustments, improving both the efficiency and consistency of adaptive planning. For example, templates can include predefined structure derivations that automatically adjust to day‐to‐day anatomical variations, minimizing real‐time contour edits and improving treatment efficiency. For example, target volumes can be defined to crop out of adjacent organs‐at‐risk automatically during adaption. These templates offer a standardized framework for consistent planning, allowing clinics using Ethos technology to maintain consistent configuration settings and optimization parameters, reducing the burden of sharing knowledge within the OART community.^1–8^

Templates, whether for Ethos or other systems, enhance treatment consistency and quality by offering a structured approach that refines techniques and reduces variability across patient populations.^9^, ^10^, ^11^, ^12^ The use of templates has become a key tool in modern RT, enabling efficient^4^, tailored treatment delivery. Sharing planning templates allows the oncology community to exchange expertise and strategies, refining practices and improving patient care.^13^, ^14^, ^15^ By disseminating viable treatment plan metrics and strategies, clinicians can refine their practices, improve the effectiveness of radiation delivery, and potentially tailor treatments more closely to patient needs. Despite these benefits, sharing and optimizing templates across institutions remains challenging.^16^, ^17^, ^18^ Traditional methods, like publications and conferences, lack the immediacy and adaptability needed for seamless integration into clinical practice. This gap in application and flexibility inhibits the potential benefits of shared treatment strategies across settings.

To bridge this gap, we developed the ADaptive rAdiotherapy Planning Template (ADAPT‐) Marketplace (https://adaptmarketplace.com), a platform for sharing Ethos compatible XML‐based templates and enabling comparison features. This user‐friendly interface supports uploading, downloading, and community‐driven innovation in adaptive radiotherapy. In this manuscript, we detail the design, development, and release of the ADAPT‐Marketplace, which facilitates the exchange of actionable knowledge and fosters collaborative advancements in OART planning. The goal of this manuscript is to describe the creation of the ADAPT‐Marketplace and its foundational use cases, establishing a framework for future evaluation of clinical impact and user adoption.

## 2 METHODS

### 2.1 Backend architecture

#### 2.1.1 Django framework for platform development

The ADAPT‐marketplace was developed and built using the Django framework. Django is a high‐level Python‐based web framework that was selected for its robustness, security, and versatility.^19^ Django, offers an array of built‐in features that ensure the development of a reliable and scalable platform. Django provides strong safeguards against common web vulnerabilities like cross‐site scripting and SQL injection, ensuring the safety of data shared on the platform. Furthermore, Django's modular design allows for rapid development and easy maintenance, which is essential for the continuous updating and scaling of the platform to meet growing user demands. Django's object‐relational mapping capabilities enable seamless integration with backend databases, allowing for efficient storage and management of XML based template data.

#### 2.1.2 XML format for template sharing

XML is the data format chosen by Varian for treatment planning templates in the Ethos system. XML's structured format allows for encoding information that is both human‐ and machine‐readable, using custom tags and hierarchical data structures to organize complex data like treatment planning parameters. This structured approach enables XML data to be easily parsed and interpreted across various systems, which is essential for cross‐system compatibility.^20^ XML templates follow a defined schema, specifying the structure, elements, and attributes required for the data, ensuring consistency and validation across different implementations. By adhering to this schema, XML templates provide a standardized format for treatment planning parameters, reducing errors and enhancing the reliability of data exchange. For treatment templates, XML's ability to store configuration settings, treatment parameters, and metadata in a standardized way facilitates access, adaptation, and customization, promoting interoperability and collaboration across clinical settings.

#### 2.1.3 Design goals of ADAPT‐marketplace

The ADAPT‐Marketplace was designed to handle treatment planning templates in XML format, ensuring compliance with all current versions of the Ethos Treatment Planning System (TPS), including v1.1 and v2.0. The platform was developed with several key design goals. First, it aims to provide an open‐source repository where clinicians and researchers can freely share and access treatment planning templates, allowing for easy information exchange and fostering collaboration across institutions. Second, it seeks to create a platform for comparison that enables users to review and benchmark their templates against those used by others in the field. Finally, it facilitates template refinement by incorporating community feedback, enabling users to collaboratively enhance existing templates and support continuous improvement in adaptive radiotherapy planning.

### 2.2 Development and testing

The development of the ADAPT‐Marketplace followed a structured process over a 1‐year period, consisting of three main phases: Requirement Analysis, which outlined design needs; Initial Design and Prototyping, which focused on development of the website; and Testing and Website Launch, which included alpha and beta testing and final deployment.

#### 2.2.1 Requirement analysis

This phase involved gathering insights from collaborators to define the platform's core functionalities and technical requirements, which was essential for ensuring that the platform would meet the expected end users’ needs. The team first conducted a feasibility assessment to evaluate whether the proposed platform was viable within the given time, budget, and resource constraints. This step involved assessing the potential challenges and risks associated with developing a web‐based repository for radiation therapy templates. Initial consultations with radiation oncologists, medical physicists, dosimetrists, and other key collaborators helped identify their specific needs, providing critical input on the features that would be most beneficial for the end users. Based on these consultations, the development team outlined the essential features of the platform, such as the ability to upload, download, and validate templates in XML format. This step also established the primary use cases for the ADAPT‐Marketplace. Finally, the technical team defined the system architecture, security protocols, and integration requirements to ensure compatibility with existing systems like the Ethos TPS while also considering scalability for future platform expansions.

#### 2.2.2 Initial design and prototyping

Once the requirements were established, the team moved into the design and prototyping phase, which involved building the initial framework of the platform, refining its features, and preparing it for testing. The focus began with user interface (UI) and user experience (UX) design, creating a user‐friendly interface that would be intuitive for clinicians to navigate. Wireframes and mockups were developed and reviewed to ensure ease of use, particularly in functions like uploading and downloading templates. A secure, scalable database structure was then designed to store the uploaded templates and associated metadata, enabling efficient management of large amounts of data while ensuring fast retrieval and processing. Using feedback from the UI/UX design phase, the team created a functional prototype, which included basic platform features such as secure login, template management, and database operations. This prototype was essential for gathering early user feedback. Based on initial user interactions with the prototype, the team refined the platform's features, incorporating improvements to key functionalities, such as the validation process for XML‐based templates.

#### 2.2.3 Testing and website launch

The final phase involved testing to ensure the platform was stable, secure, and met the users’ needs. This phase also included the launch of the platform for broader use. During alpha testing, a group of internal testers and key collaborators familiar with the intended use cases evaluated the platform to identify bugs and assess performance, focusing on ensuring that core functionalities, such as uploading and downloading templates, functioned. Feedback from the alpha testers was collected and used to make necessary adjustments to the platform, addressing potential issues before expanding testing to a larger group. Following this, beta testing was conducted with a broader group of external users from various institutions, who evaluated the platform's usability, scalability, and reliability under real‐world conditions. Feedback from beta testers provided insights into how the platform performed in diverse clinical settings, helping identify any remaining issues that needed to be addressed. Final adjustments were made based on this feedback, including bug fixes, security enhancements, and performance optimizations. The platform was then officially launched, with deployment to a live production environment, making it available to the broader radiation oncology community.

This comprehensive process ensured that the ADAPT‐Marketplace was developed with input from key collaborators, rigorously tested, and refined to meet the needs of the radiation oncology community. Each phase built upon the previous one, ensuring the platform was secure, scalable, and user‐friendly.

### 2.3 Feedback and improvements

Feedback collected through alpha (internal) and beta (external) testing, with 8 and 10 testers, respectively, was instrumental in refining ADAPT‐Marketplace. The testing involved both predefined tasks to assess specific functionalities and unscripted testing to simulate real‐world usage. Table 1 shows the evaluation tasks and a summary of the instructions. Testers, comprising a selected group of radiation oncology professionals, were asked to provide insights into the user interface, template uploading and downloading processes, and overall user experience. The testing included a post evaluation survey to collect personalized feedback.

**TABLE 1:** Step‐by‐step instructions for key tasks on the ADAPT‐marketplace platform.

Task	Instructions
Account creation	Register on ADAPT‐marketplace with your professional details, set a username and password, and confirm via the email link
Login	Log in using your credentials. Use “Forgot Password” if needed, and verify the new password works
Basic navigation	Explore the homepage, go to ‘Templates,’ and review the layout and features. Ensure all menu options work
Upload a template	Go to ‘Upload Template,’ enter required info, upload the XML file, and check that it appears in 'My Templates’
Compare a template	Select two templates from the ‘Templates’ section that you wish to compare. Use the ‘Compare’ button to view a side‐by‐side comparison of their details and specifications. Using the “add” feature, transfer an objective to another template and save as a new template
Modify a template	Choose a template you've uploaded from ‘My Templates’, click on ‘Edit’, make any desired changes to the template details or file, and save your changes. Verify the modifications are correctly reflected in the template's listing
Export a template	Select a template and use ‘Export’ to download. Confirm the file is saved correctly
Import template into ethos	Open your Ethos Treatment Planning System, navigate to the template management section, and use the ‘Import’ function to select and upload the template file you exported from ADAPT‐marketplace. Confirm the template is correctly imported and appears in the system
Complete survey	Fill out the feedback survey via the provided link, sharing your experiences and any issues

This table outlines the essential tasks for users to effectively navigate, utilize, and provide feedback on the ADAPT‐Marketplace platform, from account creation to importing templates into the Ethos Treatment Planning System.

### 2.4 Workflow and features

**FIGURE 1:**
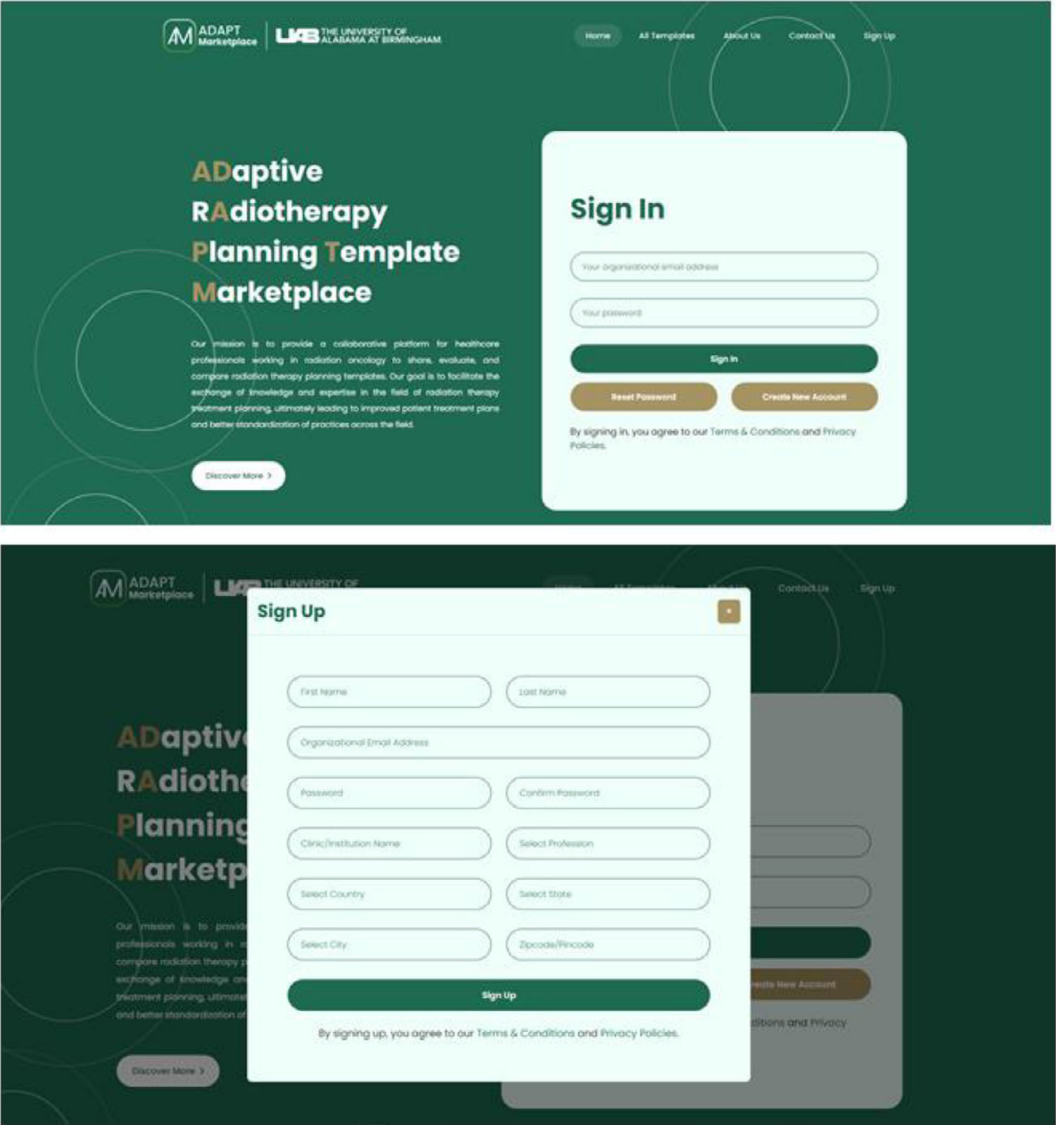
ADAPT‐marketplace login and registration interface. The login screen allows users to sign in with their credentials or create a new account by providing basic information such as name, institutional affiliation, and professional details.

**FIGURE 2:**
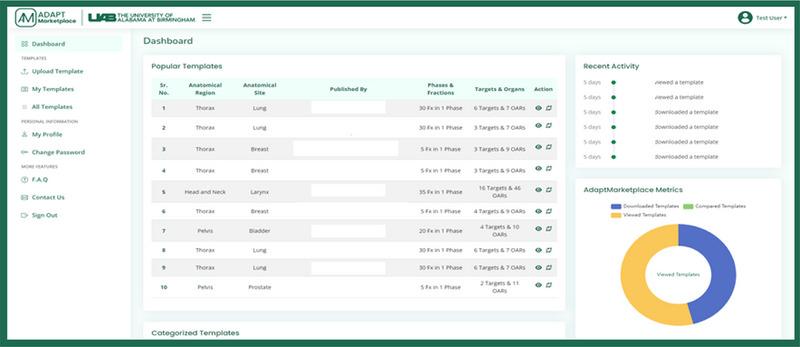
ADAPT‐marketplace dashboard and user activity metrics. The dashboard provides an overview of available templates, recent user activity, and engagement metrics, including template views, downloads, and comparisons.

**FIGURE 3:**
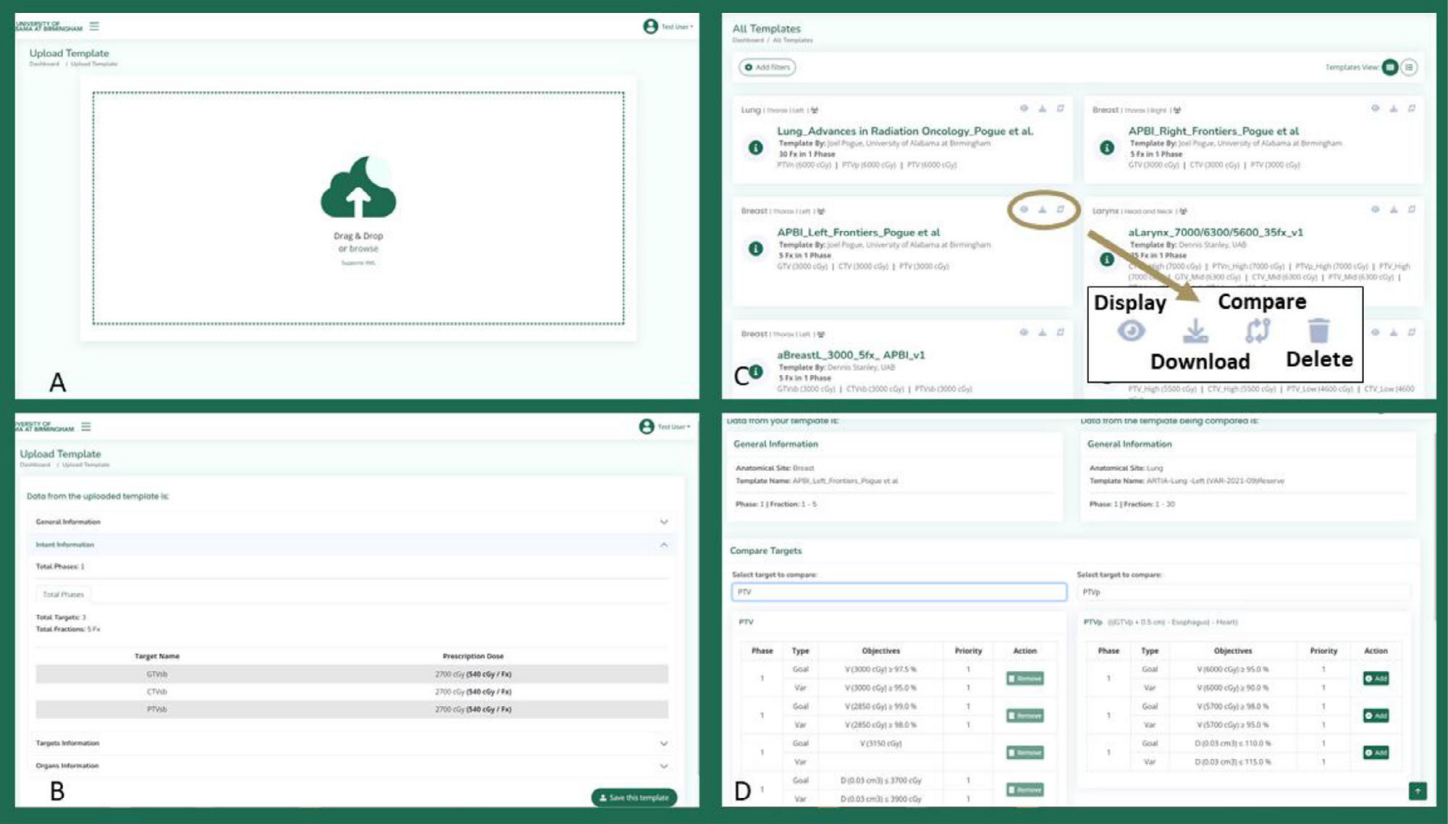
Template management and comparison tool. (A) Template upload interface, allowing users to drag and drop XML files. (B) Uploaded template details, including target names, phases, and prescription doses. (C) Template library, displaying available templates.

Figures 1, 2, 3 illustrate the interface and design features of the ADAPT‐Marketplace, highlighting its core functionalities. The login page facilitates secure access, allowing users to register by providing their name, organizational email, password, and clinic or institution demographics, creating personalized accounts for navigation (Figure 1). Upon login, the main dashboard serves as a central hub, offering an overview of popular templates, recent activities, and key marketplace metrics, such as template views, downloads, and comparisons. The dashboard enables users to upload new templates, manage existing templates, explore the full template library, adjust profile settings, and access additional features (Figure 2).

The template upload feature allows users to drag and drop or browse XML files, displaying the uploaded template's data for verification and offering the option to make it public or private (Figure 3, Panel A‐B). Uploaded templates are validated in real‐time, ensuring that the XML structure conforms to Ethos standards before the template is added to the database. Users can review templates on the platform, using filters to refine their search by anatomical site, phase structure, number of targets, and other attributes (Figure 3, Panel C). Each template listing displays optimization objectives, dosimetric targets, and associated constraints. Templates can be edited directly in the marketplace, allowing users to modify target definitions, constraints, and optimization settings as needed.

ADAPT‐Marketplace also offers a template comparison tool for side‐by‐side template evaluation (Figure 3, Panel D). Differences in phases, optimization objectives, and constraints are highlighted, giving users insight into in planning strategies variations. The comparison tool also allows users to transfer specific objectives from one template to another, creating a new customized template based on shared knowledge.

### 2.5 Post‐launch analysis

Following the official launch of the ADAPT‐Marketplace, a post‐launch analysis was conducted to assess its impact and identify opportunities for improvement. The analysis focused on key engagement metrics such as interaction patterns and user and template diversity. These metrics were used to gauge the platform's reach, usage, and effectiveness in fostering collaboration and knowledge sharing within the radiation oncology community.

#### 2.5.1 User and template engagement

We analyzed the ADAPT‐marketplace user base to understand the professional diversity and engagement levels. This included reviewing user types‐such as medical physicists, dosimetrists, and physicians – as well as their contributions. We assessed activity by examining the number of templates submitted and the geographical and institutional diversity of users. Template diversity was evaluated by analyzing the range of anatomical sites covered in the uploaded templates, from basic to complex cases. This variety highlighted the platform's capability to support different clinical needs and demonstrated its utility for both research and clinical applications.

#### 2.5.2 Interactions: views, comparisons, and downloads

To assess how users engaged with the platform, we reviewed interaction data, such as views and template comparisons and downloads. This helped determine the level of interest in the shared resources and how frequently users interacted with the available templates. By monitoring these interaction patterns, we aimed to identify areas where the platform could be enhanced to encourage more active participation and collaboration.

This analysis served as a foundation for understanding the early successes of the ADAPT‐Marketplace and helped guide strategies for future improvements, such as increasing user engagement, expanding template diversity, and enhancing the overall user experience.

## 3 RESULTS

### 3.1 Outcomes of testing phases

The testing phases, including both alpha and beta stages, played a critical role in refining the functionality and usability of the ADAPT‐Marketplace. Feedback from testers resulted in several key improvements, such as streamlined navigation, enhanced template validation processes, and refined evaluation measures. Issues like bugs in the template upload process and minor user interface glitches were identified and resolved before the platform's official launch. These iterations, based on user‐centered feedback, ensured that the platform was functional and intuitive for its intended audience of radiation oncology professionals. Key areas of feedback included requests for a more straightforward template uploading process, enhanced search functionality to quickly locate specific templates, and improved template metadata to aid in the selection process. Navigation challenges and the need for clearer instructions for new users were also addressed, enhancing the overall user experience. The responsiveness to this feedback underscored the platform's commitment to user‐centered design and practical functionality.

### 3.2 User and template engagement

Since the ADAPT‐Marketplace launched in July 2023, 65 users from 16 countries and 38 institutions, spanning both academia and industry, have registered and actively participated on the platform. The user base consists of 44 physicists, 10 dosimetrists, six physicians, three other, and two graduate students, reflecting the platform's appeal to a diverse group within the radiation oncology field. In addition to the variety of users, the platform has facilitated the sharing of more than 50 treatment‐planning templates, most of which are publicly available. These templates cover a wide range of anatomical treatment sites, including the pelvis, thorax, head‐and‐neck, and abdomen. The complexity of the templates ranges from straightforward cases to more advanced plans. For example, one template includes four simultaneous integrated boost levels with different prescription doses, 15 organs at risk, and multiple individual dose objectives, showcasing complex dosimetric considerations and supporting both routine clinical use and educational purposes. The median user has contributed 0.6 publicly available templates with the most active user having contributed 8 downloadable templates.

### 3.3 Interactions: views, compares, and downloads

For this analysis, user interactions are defined as actions that involve engaging with a template, including viewing, comparing, or downloading. Users who browse templates but do not engage with specific ones (through views, comparisons, or downloads) are not included in the interaction count. Interactions performed during the alpha and beta testing phases were also excluded, as the system was reset before the official launch. Table 2 summarizes user interactions on the ADAPT‐Marketplace at two time points: 6 and 12 months after launch. Interactions are categorized into three types – template views, downloads, and comparisons with save/download actions – highlighting an overall increase in engagement over time. The total number of interactions grew from 162 at 6 months to 395 at 12 months, reflecting the platform's expanding usage and active participation.

**TABLE 2:** User interactions on the ADAPT‐Marketplace measured at 6 and 12 months post‐launch (cumulative).

	Interaction frequency	
Interaction type	6 months	12 months (cumulative)
Template – View only	78	219
Template – Download	48	97
Template – Compare and save/download	36	79
Total interactions	162	395

This table summarizes the frequency of user interactions with templates on the ADAPT‐Marketplace platform over 6‐month and 12‐month periods, including view‐only actions, downloads, and template comparisons with save/download functionality, highlighting a significant increase in total interactions over time.

### 3.4 System performance and security

The performance of ADAPT‐marketplace, hosted on a scalable and secure server infrastructure, has met expectations in terms of reliability and speed. The use of the Django web framework has facilitated efficient data management and user interaction, supporting a smooth and responsive experience for users. Performance metrics indicate quick load times for pages and templates, even under conditions of high user access, ensuring that the platform remains accessible and functional for global users across different time zones. The platform's security measures, including data encryption and compliance with healthcare data protection standards, have ensured the safe and confidential handling of user data and template information. Continuous monitoring and periodic security assessments are in place to safeguard against potential vulnerabilities, maintaining user trust and platform integrity.

## 4 DISCUSSION

### 4.1 Collaborative potential of ADAPT‐marketplace

The ADAPT‐Marketplace has created a platform for the rapid exchange of treatment planning knowledge within the CBCT‐guided Adaptive Radiotherapy community. By enabling the sharing and optimization of Ethos‐based templates, it supports collaborative innovation and aims to enhance patient care. As a central hub for optimized treatment planning, the platform helps address disparities in quality between institutions, allowing radiation oncologists, physicists, and dosimetrists to benefit from a growing knowledge repository.^21^ Engagement metrics show strong interest across diverse geographical and institutional settings, indicating significant growth potential.

A key feature of ADAPT‐Marketplace is the template comparison tool, which allows users to analyze multiple templates side by side, gaining insights into optimization objectives, structural elements, and dosimetric considerations. This tool not only integrates best practices into new templates but also serves as a key driver for expertise exchange, helping clinicians refine treatment approaches based on shared knowledge. The comparison tool enhances collaborative learning by allowing users to analyze templates side by side, integrate best practices, and apply shared insights directly to their own clinical workflows. In the first 6 months after launch, template comparisons were a major user activity, highlighting the trend toward collective learning. As the user base expands, these interactions are expected to grow, providing more opportunities for shared expertise and enhanced treatment planning. Future developments will focus on improving the comparison feature and encouraging greater user participation.

Another important consideration for template sharing through ADAPT‐Marketplace involves the use of RapidPlan models. RapidPlan models can be used to predict DVHs and guide optimization based on prior data and clinical experience, which can influence plan quality.^16^, ^22^ While RapidPlan models can be paired with Ethos templates, they are not exported automatically with the template. Therefore, a clinic importing a template from ADAPT‐Marketplace that was originally created with an associated RapidPlan model would need to import the corresponding model to achieve similar plan quality. Without the linked RapidPlan model, the imported template may not deliver the same dosimetric performance, leading to deviations in plan quality when sharing tempaltes. Addressing this limitation may require developing guidance on how to handle RapidPlan dependencies when sharing templates across institutions. Other repositories such as ORBIT‐RT^15^ and Varian's Medical Affairs site may provide complementary hosting options for RapidPlan models, which could support broader template sharing and reproducibility.

### 4.2 Enhancing research productivity and clinical practice

The communal nature of ADAPT‐Marketplace provides opportunities to enhance both research productivity and clinical practice. By enabling the dissemination of best practices, the platform supports institutions with limited access to advanced planning expertise, particularly in resource‐constrained settings. This approach fosters collective treatment solutions, reduces variability in care, and is especially beneficial for low‐resource institutions. Evaluating data on template efficacy and user feedback offers insights into the application and performance of ART templates across various treatment sites and patient demographics. The platform facilitates the development of expertise, allowing independent institutions to benefit from knowledge shared by academic and industry experts.

ADAPT‐Marketplace also lays the groundwork for future research. With growing users and templates, it enables comparative effectiveness studies, machine learning‐driven optimizations, and the creation of standardized metrics for evaluating plans. These avenues help establish best practices, refine workflows, and improve outcomes across diverse disease sites. By leveraging its collective expertise, the platform fosters a collaborative environment where shared challenges are addressed through practical innovation.

Despite early promise, challenges remain. The platform's sustainability relies on continued user engagement and contributions. Building a strong community of users contributing high‐quality templates requires ongoing community building and incentives. Additionally, variability in institutional practices necessitates rigorous validation of externally sourced templates before clinical use, highlighting the need for clear guidelines and collaboration. Addressing these issues is vital to maintaining user trust and ensuring the platform's long‐term success.

### 4.3 Limitations and challenges

While ADAPT‐Marketplace provides a valuable resource for the radiation oncology community, it is currently limited to Ethos templates. As a result, the user base is inherently constrained to institutions using Ethos. As of the date of this writing, there are approximately ∼150 Ethos systems installed worldwide, with 50 in the United States. Although the number of Ethos installations is increasing, this creates a ceiling on the potential growth of the ADAPT‐Marketplace user base. Expanding the platform's compatibility to other treatment planning systems could broaden its reach and impact.

Additionally, the platform's functionality is dependent on the template formatting defined by the vendor. If Varian modifies the template structure or changes the way the optimization engine interprets dose goals, earlier templates may no longer be compatible. For example, the transition to Ethos 2.0 involved changes in dose calculation algorithms that affected template behavior. Such changes could impact the performance of existing templates and limit their future usability. Providing users with guidelines on how to adapt templates to platform upgrades may help mitigate this risk.

System performance and reliability are critical to the platform's success, and we understand that uptime is essential for maintaining user trust and engagement. Since launch, we have experienced occasional isolated downtime events caused by a variety of factors related to platform configuration and hosting infrastructure. For example, one outage occurred when a security certificate expired without triggering a renewal notification, and another instance involved the hosting service failing to restart properly after scheduled maintenance. While we did not have continuous uptime monitoring in place during these early incidents, we have since implemented real‐time monitoring, automated restarts, and proactive notification systems to minimize the risk of future disruptions. Ensuring platform stability remains a top priority, and we continue to learn not only from these technical challenges, but also from the broader experience of operating and maintaining a web‐based platform at scale. As the platform grows, so too does our understanding of the infrastructure and practices needed to support a reliable, accessible tool for the radiation oncology community.

Finally, while early engagement metrics suggest growing interest in the ADAPT‐Marketplace, this manuscript does not include direct evidence of clinical implementation or outcomes resulting from shared templates. Metrics such as logins, downloads, and template views are useful for characterizing platform activity but are limited in their ability to validate real‐world utility. Understanding how users incorporate templates into clinical workflows – along with the resulting impact on efficiency, plan quality, or patient outcomes – represents a critical next step. To support this, we are developing infrastructure to collect implementation feedback from users, including optional case reporting and embedded surveys. These efforts are ongoing and will form the basis of future work aimed at evaluating the clinical impact of template sharing.

## 5 CONCLUSION

By providing a centralized platform for template sharing and collaboration, ADAPT‐Marketplace has the potential to enhance research productivity, facilitate knowledge exchange, and increase the utilization of online adaptive RT with the goal of improving patient outcomes. To date, ADAPT‐Marketplace has 65 registered users from 16 countries and 38 institutions, both academic and industrial. Treatment sites covered by uploaded templates include pelvis, thorax, head‐and‐neck, and abdomen. Looking ahead, expanding the range of treatment sites and diversifying the user base will broaden the platform's impact, bringing in new perspectives and expertise across the radiation oncology community. Integrating AI and machine learning could further enhance template design by enabling real‐time adaptation, streamlining the planning process, and improving treatment precision and efficiency.

## CONFLICT OF INTEREST STATEMENT

Richard A. Popple has contracts and honoraria for presentations with Varian Medical Systems and receives royalties for a patent licensed with UAB Research Foundation to Varian Medical Systems. John B. Fiveash reports grants from Varian Medical Systems unrelated to the present study. Dennis N. Stanley receives consulting fees, payment or honoraria for lectures, presentations, speaker bureaus, manuscript writing, or educational events, and support for attending meetings and/or travel from Varian Medical Systems as an Educational Consultant. The other authors declare that they have no known competing financial interests or personal relationships that could have appeared to influence the work reported in this paper.

## Data Availability

Research data are stored in an institutional repository and can be shared upon request to the corresponding author.
